# Stability analysis of mixtures of mutagenetic trees

**DOI:** 10.1186/1471-2105-9-165

**Published:** 2008-03-26

**Authors:** Jasmina Bogojeska, Thomas Lengauer, Jörg Rahnenführer

**Affiliations:** 1 Max-Planck-Institut für Informatik, Stuhlsatzenhausweg 85, 66123 Saarbrücken, Germany; 2 Department of Statistics, University of Dortmund, Vogelpothsweg 87, 44221 Dortmund, Germany

## Abstract

**Background:**

Mixture models of mutagenetic trees are evolutionary models that capture several pathways of ordered accumulation of genetic events observed in different subsets of patients. They were used to model HIV progression by accumulation of resistance mutations in the viral genome under drug pressure and cancer progression by accumulation of chromosomal aberrations in tumor cells. From the mixture models a genetic progression score (GPS) can be derived that estimates the genetic status of single patients according to the corresponding progression along the tree models. GPS values were shown to have predictive power for estimating drug resistance in HIV or the survival time in cancer. Still, the reliability of the exact values of such complex markers derived from graphical models can be questioned.

**Results:**

In a simulation study, we analyzed various aspects of the stability of estimated mutagenetic trees mixture models. It turned out that the induced probabilistic distributions and the tree topologies are recovered with high precision by an EM-like learning algorithm. However, only for models with just one major model component, also GPS values of single patients can be reliably estimated.

**Conclusion:**

It is encouraging that the estimation process of mutagenetic trees mixture models can be performed with high confidence regarding induced probability distributions and the general shape of the tree topologies. For a model with only one major disease progression process, even genetic progression scores for single patients can be reliably estimated. However, for models with more than one relevant component, alternative measures should be introduced for estimating the stage of disease progression.

## Background

A grand challenge in biomedical research is improving diagnosis and therapy for various diseases by using genetic profiles from the patients. This can enable *personalized medicine*, where the therapy of a patient is influenced not only by the results of the conventional medical analysis, but also by the analysis resulting from the values of the individual genetic composition.

The mutagenetic trees mixture model introduced by Beerenwinkel *et al*. [1] provides an interpretable probabilistic framework for modeling multiple paths of ordered accumulation of permanent genetic changes. The model describes several alternative pathways of disease development, each in a different mixture component. Datasets used for estimating the mixture model consist of patterns of genetic events observed for single patients. From the mixture model, a genetic progression score (GPS) can be computed for each pattern [2] that incorporates correlations among genetic events and mean time intervals between their occurrences. The GPS gives an estimate of the progression of the disease process and can be used for specifying therapies or estimating survival times of the patients [2].

Both the mixture model itself and derived features like GPS values have been proven to improve the interpretation of disease progression and estimation of survival times in the context of two different diseases, namely HIV [1] and cancer [2]. However, due to the complexity of the mixture model, it is important to analyze the stability of the estimation process and of features that are calculated from the model.

In this manuscript, we present a simulation study for inspecting the stability of estimated mutagenetic trees mixture models. We compare simulated true mixture models with models fitted to observations drawn from these true models. The stability analysis refers to GPS values as well as to other features of the mixture model like induced probability distributions or tree topologies. A *bootstrap method *[3] is used for a more detailed analysis of the variability of the GPS values and for deriving GPS confidence intervals.

The EM-like learning algorithm used for estimating mutagenetic trees mixture models finds a starting solution by an initial clustering step. We extend the fitting procedure given in [1] by specifying different initial assignments of the responsibilities, i.e. a different starting solution for mixture model fitting. We introduce the *diversity parameter d *that describes the diversity of the initial tree components. Based on simulated models we specify an optimal value for *d *with regard to the reliability of the final estimated models.

### HIV drug resistance

The mutagenetic trees mixture model can be used for modeling HIV evolution as a process of accumulation of mutations in the viral genome under drug pressure. The replication rate of the HI-Virus is extremely high. Under therapy, genetic mutations enable the virus to develop drug resistant mutants. The evolution of HIV *drug resistance *is conveyed by the erroneous reverse transcription during virus replication, the large diversity of the HIV population, and the natural selection of the fittest mutant under drug pressure. This results in a dynamic and highly adaptive virus population.

In this work we focus on HIV-1 drug resistance to the nucleoside reverse transcriptase (RT) inhibitor *zidovudine*. The most prevalent mutations in the HIV-1 genome that rise under zidovudine are M41L, D67N, K70R, L210W, T215F/Y, and K219E/Q [4,5]. Typically, K70R and T215F/Y are the first mutations to occur [6]. The set of mutations 215F/Y, 41L, and 210W, also known as 215 – 41 pathway, occurs together. The same holds for 70R and 219E/Q (70 – 219 pathway). In our experiments we use the dataset from the Stanford HIV Drug Resistance Database [7] that comprises genetic measurements of 364 HIV patients treated only with the drug zidovudine. The data contains the six classical major zidovudine resistance mutations mentioned above.

### Evolutionary tree models

In this section, we present the mutagenetic trees mixture models as means to model disease evolution. Furthermore, we describe the genetic progression score (GPS) derived from these models used for quantifying the stage of the disease.

### Mutagenetic trees mixture models

The mutagenetic trees mixture model is a probabilistic model that can describe multiple pathogenetic routes of ordered genetic mutations in disease progression. A single mutagenetic tree is a weighted directed tree in which the genetic events are represented by nodes and weights on the edges correspond to the conditional probability of the child event happening given that the parent event has occurred. This tree structure provides a probabilistic model that annotates evolutionary paths of disease progression. A disadvantage of this model is that many subsets of genetic events cannot be explained by a single tree, since it can only represent those subsets of events which include events together with all their predecessors in the tree. All other subsets of events have likelihood zero in the probability distribution generated by a single tree model. A single tree also often fails to capture all diverse pathogenetic routes that can occur in disease progression. Formal details of the definition and estimation of single trees are presented in [8].

The mutagenetic trees mixture, proposed by Beerenwinkel *et al*. in [1], provides a probabilistic model that can capture multiple evolutionary processes conducting the disease evolution, each of them in a separate mixture component. We consider *K *mutagenetic trees *T*_*k*_, *k *= 1,...,*K*, on the set of vertices *V *= {0,...,*l *- 1} denoting *l *events with the null event *r *as root. The root comprises events that has initially occurred in all samples and accounts for the therapy naive patients. Moreover, it represents the starting point of disease progression. The tree *T*_1 _has a star-like topology and describes the genetic events as being independent of each other given the initial null event. According to the probability distribution it induces, the star tree assigns positive probability to all possible 2^*l*-1 ^patterns generated by a given set of *l *genetic events [1].

Formally, a mutagenetic trees mixture model *M *is a weighted sum of *K *mutagenetic trees $M=\sum_{k=1}^{K}\alpha_{k}T_{k}$, where *α*_*k *_∈ [0, 1] and $\sum_{k=1}^{K}\alpha_{k}=1$. Every subset of genetic events, determined by the pattern *x*, has positive probability of being generated, given by:

$$\mathrm{Pr}(x|M)=\sum_{k=1}^{K}\alpha_{k}\mathrm{Pr}(x|T_{k}).$$

Let *V *be the set of vertices corresponding to the genetic events, and let *E *= {(*u*, *v*) | *u*, *v *∈ *V*} be a set of edges. For all edges *e *= (*u*, *v*) ∈ *E*, the edge weights indicate the conditional probability that the event *v *appears given that the event *u *has occurred, and are determined with the mapping *p*: *E *→ [0, 1], such that:

*p*(*e*) = Pr(*X*_*v *_= 1 | *X*_*u *_= 1).

The root vertex denoted by *r *is the node 0 and specifies the initial null event, such that Pr(*X*_*r *_= 1) = 1. The connected branching *T *= (*V*, *E*, *p*) formally captures the notion of a *mutagenetic tree*.

Let Ω = {0, 1}^*l *^be the set of all possible patterns of genetic events of length *l *and *x *be an observation defined by the subset *S *⊆ *V *of events that have occurred. Each observation *x *is a binary vector with ones indicating occurrence of mutations. For example, if the pattern (0, 0, 1, 0, 1) is associated with a specific patient this means that the genetic mutations 3 and 5 have occurred in the respective sample. The mutagenetic tree *T *= (*V*, *E*, *p*) induces a probability distribution on the set Ω. Accordingly, if there exists an edge subset *E' *⊆ *E*, such that *S *is the set of vertices reachable from the root in the induced tree *T' *= (*V'*, *E'*, *p*), then the probability that the tree *T *generates the pattern *x *is:

$$\mathrm{Pr}(x|T)=\prod_{e\in E^{\prime }}p(e)\cdot \prod_{e\in S\times (V\backslash S)}\left(1-p(e)\right).$$

Otherwise, the mutagenetic tree *T *cannot generate the pattern *x*, i.e. Pr(*x *| *T*) = 0.

Given the number of tree components *K *and a finite sample of *N *observations *D *= {*x*_1_,...,*x*_*N *_} of the binary vector *X *= (*X*_1_,...,*X*_*l*_) that indicates occurrence of subsets of genetic events, the mixture model can be estimated as follows. Assuming that for each sample the tree component from which that sample was generated is known, one can easily reconstruct the mixture model by using Edmonds' branching algorithm [9]* K *times on the respective observation sets. For large number of samples Edmonds' branching algorithm reconstructs the original mutagenetic tree with very high probability [9]. Since one does not know from which tree each sample was generated, one has to estimate it from the data. The goal is to find trees *T*_1_,...,*T*_*K *_and mixture parameters *α*_1_,...,*α*_*k *_that maximize the log-likelihood of the data. Having a mixture model, a standard procedure for maximum-likelihood estimation is the EM algorithm [10].

**Figure 1:**
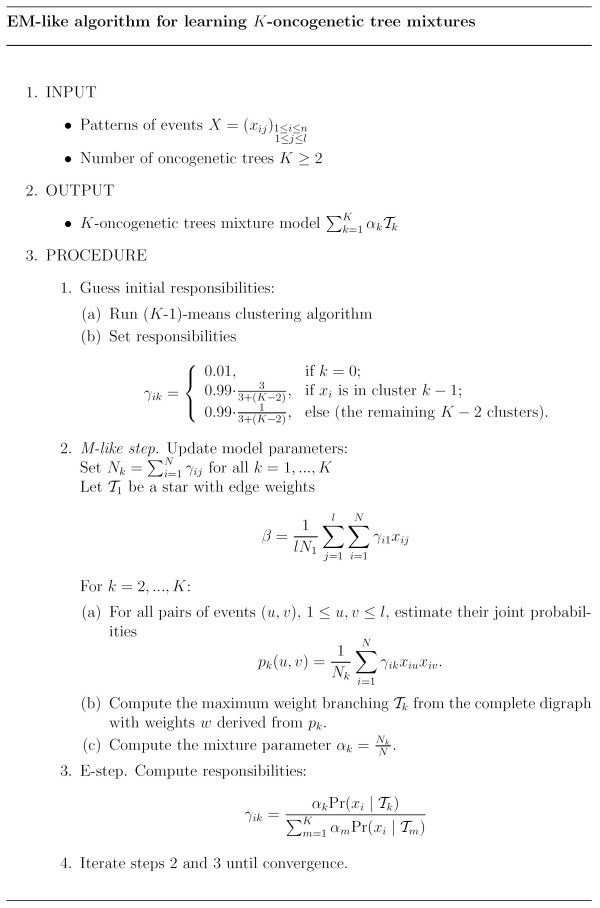
**EM-like algorithm for learning mutagenetic trees mixture models**. Detailed EM-like algorithm for fitting K-trees mutagenetic mixture models from a given dataset, adapted from Beerenwinkel *et al*. [1].

Let Δ_1_,...,Δ_*K *_be binary random variables where Pr(Δ_*K *_= 1) = *α*_*k*_. The *responsibility *of the *k*-th tree component *T*_*k *_for the *i*-th observation *x*_*i *_is the probability that *x*_*i *_was generated from *T*_*k *_given the mixture model *M*: *γ*_*ik *_= Pr(Δ_*k *_= 1 | *x*_*i*_, *M*). The EM-like algorithm for fitting mutagenetic trees mixture models is briefly described below. A detailed description is presented in Figure 1. The algorithm is only an "EM-like"-algorithm, since the tree reconstruction step with Edmonds' algorithm does not provide an exact maximum likelihood estimate. In practice, the solution of Edmonds' algorithm is close to the maximum-likelihood solution.

1. *Guess initial responsibilities: *Run (*K*-1)-means clustering algorithm on the given dataset and assign samples to the corresponding (*K *– 1) clusters.

2. *Maximization-like step: *Estimate the *K *tree components *T*_1_,...,*T*_*K *_from all patterns weighted with their responsibilities and compute the mixture parameters *α*_1_,...,*α*_*k*_.

3. *Expectation step: *Recompute the responsibilities of the samples by using the tree components estimated in the previous step.

4. Iterate the two previous steps until convergence.

The solution of an EM algorithm depends on the initial values used for the responsibilities. The mixture model tries to capture diverse paths of ordered genetic changes present in the data. Furthermore, only a small fraction of the dataset, that cannot be mapped to the nontrivial components of the mixture, should be mapped to the star component. The rest should be assigned to the nontrivial trees of the mixture model. In [1], the starting solution is determined by running a (K – 1)-means clustering algorithm on the set of observed patterns *D *by using the squared Euclidian distance as dissimilarity measure [11]. Here we propose initial assignments of the responsibilities depending on a *diversity parameter d*. This parameter controls the diversity of the initial tree components comprising the mixture model and, as a consequence, also the diversity of the components in the EM solution. The optimal value of *d *= 3 is chosen as a result of the simulation study presented in the Results section. This value is a good compromise between diversity of nontrivial branchings and quality of the fitted model.

**Figure 2:**
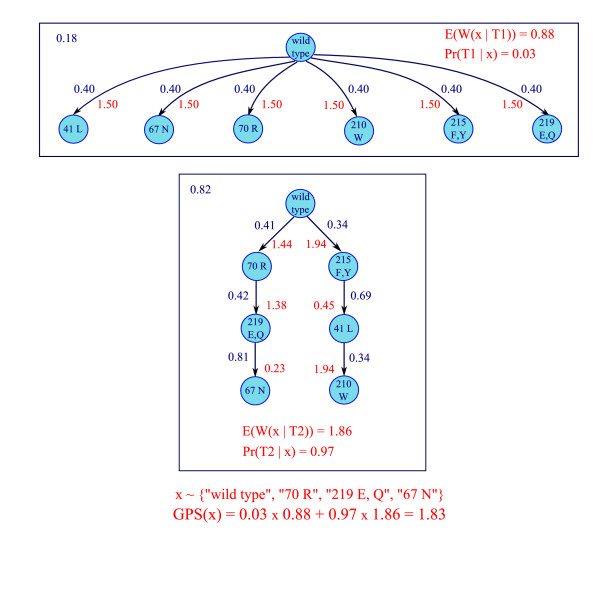
**Mutagenetic trees mixture model with two components**. Timed mutagenetic trees mixture model estimated on the HIV dataset. The responsibility of the nontrivial tree component is 0.82. A sample is generated from the noise component with probability 0.18. The nodes represent the genetic events and the labels of the edges depicted with dark blue color are the conditional probabilities between the events. The nontrivial branching of the mixture model shows the two typical pathways 70 – 219 and 215 – 41 that develop under zidovudine monotherapy. Exponential distributions are assumed for the time difference between the occurrences of the child and parent events on all edges and for the sampling time (with mean sampling time equal to 1). The timed model then is obtained by mapping the edges with the expected waiting times of occurrence of the child events (red color), given that the parent events has occurred. The GPS of the pattern *x*, which specifies the occurrence of the subset of events {0, 70*R*, 219*E/Q*, 67*N*}, is calculated using the timed mutagenetic trees mixture model and formula (1).

The EM-like algorithm assumes that the number of trees *K *is known. The algorithm is efficient enough to estimate this model parameter in a cross-validation framework, as proposed in [1]. Yin *et al*. [12] introduce a modified *Bayesian Information Criterion *(BIC) for estimating the number of trees. The modified BIC incorporates a similarity measure for estimating the structural redundancy between tree components in the penalization term of the standard BIC. A mutagenetic trees mixture model estimated for HIV patients is illustrated in Figure 2. The model complies with the main experimental results about HIV-1 zidovudine resistance.

### Genetic progression score (GPS)

Considering the tree structure of the components of the mixture model, under some additional assumptions waiting times can be mapped on the tree edges. Consequently, a progression score that incorporates correlations among events and time intervals among occurrences of events can be associated to the mixture model as proposed in [2].

The timed mixture model can be obtained by assuming independent Poisson processes for the occurrence of events on the edges of each mixture component and for the sampling time of the disease. The *sampling time *denotes the time interval between the onset of the disease and its discovery or analysis. Let the waiting time *T*_*i *_of the *i*-th event denote the time difference between the occurrences of its parent event *pa*(*i*) and the event itself. Let *T*_*i *_and the sampling time *T*_*S *_have exponential distributions with parameters *λ*_*i *_and *λ*_*S*_, respectively. The conditional probability *p*_*i *_assigned to the edge (*pa*(*i*), *i*) that enters the *i*-th event can be calculated as *p*_*i *_= *λ*_*i*_/(*λ*_*i *_+ *λ*_*S*_). In this framework the expected waiting time E(*i*) for an event *i *is given as follows:

$$\text{E}(T_{i})=\frac{1}{\lambda_{i}}=\frac{1-p_{i}}{p_{i}}\frac{1}{\lambda_{S}}=\frac{1-p_{i}}{p_{i}}\text{E}(T_{S}).$$

Typically, the onset of the disease is not known for single patients. Thus *λ*_*S *_cannot be derived from the data and the waiting times E(*T*_*i*_) on the edges cannot be expressed in timescale of the process of genetic progression. Therefore the mean sampling time is normalized to 1, i.e. E(*T*_*S*_) = 1, and unitless waiting times are mapped on the edges of the trees of the mixture model.

It is easy to calculate the expected waiting time for a single event *i *and map it to the corresponding edge (*pa*(*i*), *i*). However, an explicit closed formula for computing the expected waiting times for an arbitrary pattern *x*_*i *_of genetic events cannot be derived. Using the timed mutagenetic trees mixture model *M *the expected waiting times of arbitrary patterns of events can be obtained by simulating the waiting process along the edges of each tree component and using the probability distribution induced by the mixture model.

When simulating the waiting process along a single tree from the mixture with sufficiently large number of simulations (≥ 10^6^), first, waiting times for the events *i *on the edges (*pa*(*i*), *i*) are drawn from exponential distributions with parameters *λ*_*i *_= *p*_*i*_/(1 - *p*_*i*_). Since the tree structure captures the order in which the genetic abberations occurred, the waiting times of subsequent events along the tree topology are added.

Considering the simulation of the waiting process for the *k*-th tree component *T*_*k*_, the expected waiting time for the subset of events *x*_*i*_, denoted with ${\text{E}}_{T_{k}}$(*W *(*x*_*i*_)), is the average of all waiting times at which the pattern *x*_*i *_was observed. Finally, the expected waiting time of the pattern *x*_*i *_with respect to the given mixture model, referred to as the GPS of the pattern, is a weighted sum of the expected waiting times of *x*_*i *_with respect to each of the *K *mixture components. The weights are the responsibilities of the respective tree components for the pattern of events *x*_*i*_.

Formally, the GPS of the pattern *x*_*i *_with respect to the mixture model *M *is given by

(1) $$\begin{array}{lll} \text{GPS}(x_{i}) & = & {\text{E}}_{M}(W(x_{i}))=\sum_{k=1}^{K}\mathrm{Pr}(T_{k}|x_{i})\cdot {\text{E}}_{T_{k}}(W(x_{i}))\\ & = & \sum_{k=1}^{K}\gamma_{ik}\cdot {\text{E}}_{T_{k}}(W(x_{i})). \end{array}$$

The computation of the GPS for the pattern *x *= {0, 70*R*, 219*E/Q*, 67*N*} from the HIV dataset is depicted in Figure 2.

## Results and Discussion

We present the results of a stability analysis for mutagenetic trees mixture models and features derived from such models. In short, the stability analysis was performed with the following approach. First, a "true" mutagenetic trees mixture model is drawn uniformly at random from the model space, and a data sample is drawn from this true model. Then, a mutagenetic trees mixture model is fitted to the sample. The quality of the fitted model is assessed by comparing its quality with the quality of a sufficient number of random mixture models sampled uniformly from the mixture model space.

The quality of a model is computed with respect to a specified model feature. A similarity measure has to be defined for comparing two tree mixture models based with respect to this feature. The similarity between the true and the fitted model is then compared to the similarity between the true and the random models. A p-value is obtained as the percentage of cases, in which the true model is closer to a random model than to the fitted model. Similarity is defined in various ways, comparing the probability distribution induced by the model, the topologies, and the GPS values calculated from the models.

In the publication introducing mutagenetic trees mixture models [1], the initial step of the EM-like algorithm for fitting a K-mutagenetic trees mixture models is determined by first running a (K-1)-means clustering algorithm on the set of observed patterns. Then, all observations in one cluster are assigned to one component with probability 0.5 and to the other components with equal probability. Here, we introduce a diversity parameter *d *that determines the softness of this initial assignment. We show that the setting of the diversity parameter influences the stability of the mixture models.

The Methods section below introduces the similarity measures used for comparing mutagenetic trees mixture models and gives a detailed description of the simulation setup used in the stability analysis.

In the following, first, the influence of the initial cluster assignments on the model diversity is discussed. Then, the results of the detailed stability analysis are presented. Finally, the results of a bootstrap analysis for estimating confidence intervals of the GPS values is presented.

### Influence of initial clustering on diversity between model components

In [1], the starting solution for the EM-like algorithm is determined by running a (K – 1)-means clustering algorithm on the set of observed patterns and subsequent soft assignment of clusters to model components.

Let *γ*_*ik *_denote the responsibility of the *k*-th mixture model component for generating the *i*-th observation.

The initial responsibilities in [1] are defined by

(2) $$\gamma_{ik}=\{\begin{array}{ll} \frac{1}{2}, & \text{if }x_{i}\text{ is in cluster }(k-1);\\ \frac{1}{2(K-1)}, & \text{else}. \end{array}$$

We propose different assignments of the initial responsibilities as follows.

(3) $$\gamma_{ik}=\{\begin{array}{ll} 0.01, & \text{if }k=0;\\ 0.99\cdot \frac{d}{d+(K-2)}, & \text{if }x_{i}\text{ is in cluster }(k-1);\\ 0.99\cdot \frac{1}{d+(K-2)}, & \text{else }(\text{the remaining }(K-2)\text{ clusters}). \end{array}$$

The diversity parameter *d *controls the softness of the initial assignment. In order to choose an optimal value for *d *we performed two different simulations. The first analysis compares the topologies of the nontrivial tree components within a mixture model. This reflects the diversity of the paths of disease progression captured by the mixture model.

**Figure 3:**
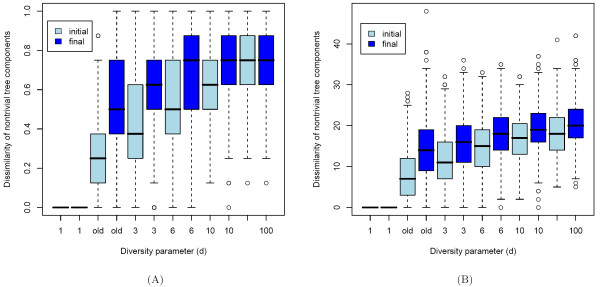
**Analysis of the diversity parameter**. A) Diversity of the nontrivial components in the initial (light blue color) and final (blue color) mixture models for different cluster assignments. The tree comparisons in the experiments are performed with the similarity measure (5) based on the sum of the number of different edges. The larger *d *is in (3), the more diverse are the nontrivial components in the initial and the final models. B) Diversity analysis as in Figure 3A, but with the similarity measure (6) based on the sum of the number of different edges and the *L*_1 _distance of the vectors containing the levels of the events.

We usually repeat the simulation procedure described in the Methods section 500 times using either the previous cluster assignments (2) or the new assignments (3) for diversity parameter settings *d *∈ {1, 3, 6, 10, 100}. The objective of this analysis is to see how the initial assignments of the responsibilities determined by the (*K *– 1) – means clustering [11] affect the diversity of the topologies of the nontrivial branchings of the final model. For this purpose we calculate the diversity of model branchings in the initial and in the final mixture model. The initial model is obtained from fitting tree components to the clustering results (the starting point in the model search space), and the final model is obtained from the EM-like search algorithm [1,10]. The results for the two different similarity measures for comparing tree topologies (5) and (6) are depicted in Figure 3A and Figure 3B, respectively.

Both figures show that the larger *d *is, the more diverse are the nontrivial components in the initial and the final model. For *d *= 1 the nontrivial components in the models (initial and final) are equal. In what follows, we consider only the tree topology similarity measure (6).

### Influence of initial clustering on stability

Next, we evaluated the quality of the fitted models depending on the different cluster assignments. The notion "quality of the estimated mixture models" refers to the goodness of fit of the probability distributions induced by the mixture models, of the fitted tree topologies, and of the fitted GPS values. For exploring these features we performed simulations using the simulation setup described in the Methods section, using both the old clustering (2) and the new one (3) with *d *∈ {3, 6, 10, 100}. For these simulations *K *= 3 model components were used. For all different cluster assignments the probability distributions were estimated with high quality, documented by p-values smaller than 0.05 in all simulation iterations.

**Figure 4:**
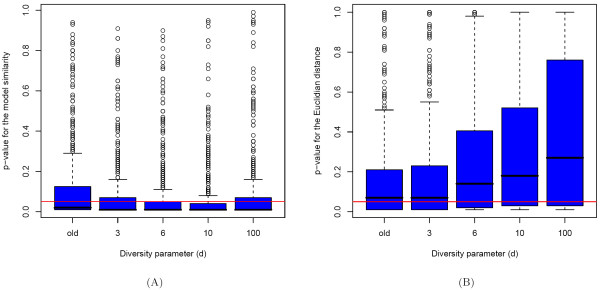
**Stability of tree topology and GPS for different cluster assignments**. A) Significance of the similarity between the tree topologies of the true and the corresponding fitted mixture models. The red line depicts the threshold p-value = 0.05. The cluster assignments given by (3) with *d *∈ {3, 6, 10} achieve the best estimation of the tree topologies. B) Significance of the similarity between the GPS vectors resulting from the true and the corresponding fitted mixture models. The red line depicts the threshold p-value = 0.05. The previous clustering (see [1]) and the new one (see formula (3)) with *d *= 3 achieve the best results.

The experimental results rendering the quality of the fitted tree topologies are shown in Figure 4A, where we used the similarity measure (6) for comparing tree topologies. Boxplots of p-values are depicted, that quantify the superiority of the fitted model over random models. It can be seen that the new cluster assignments with *d *∈ {3, 6, 10} lead to more significant results than the previous cluster assignments (2). We confirmed this observation by applying a two-sided test for equality of the proportions of p-values that are less than or equal to 0.05. Only for *d *= 100 the improvement of the new cluster assignments with respect to the previous cluster assignments is not significant at the significance level 0.05.

The stability of GPS values strongly depends on the tree topologies and on the conditional probabilities assigned to the tree edges. The simulation results for the different cluster assignments are shown in Figure 4B. Here, the Euclidian distance is used for comparing the GPS vectors. The best results are obtained for the old clustering method and for *d *= 3 with the new clustering method. Larger values for the diversity parameter *d *are too extreme and make the GPS estimation more unstable.

To summarize, using the cluster assignments defined by (3) with *d *= 3 is a good compromise between diversity of the nontrivial branchings and quality of the fitted branchings. The new cluster assignments achieve the same fitting quality regarding the probability distributions and the GPS values as the previous cluster assignments introduced in [1]. However, they provide higher diversity of the nontrivial tree components and significantly better estimation in terms of the topology of the branchings of the fitted models (see Figure 4A).

In the final estimated mixture model the fraction of the dataset that can be mapped only to the noise component should be as small as possible. Thus it is promising to start the EM-like algorithm from a point in the model space with this property. Therefore, in formula (3), we initially assign each observation to the noise component with the small probability 0.01. For the more extreme value of 0.001 we obtained comparable results.

### Stability of the probability distribution

The mutagenetic trees mixture models generate a probability distribution on the set of all possible patterns for a given number of genetic events *l *[1]. The EM-like algorithm [10] used for learning the mixture model from a given set of patterns maximizes the log-likelihood of the patterns in terms of this probability distribution. We performed a stability analysis using the simulation setup described in the Methods section. We used the Cosine distance, the *L*_1 _distance and the Kullback-Leibler divergence for calculating the similarity between the probability distribution induced by the "true" model and the one induced by the fitted model.

The simulation results are similar for *K *= 2 and for *K *= 3 branchings in the mixture models (data not shown). For reasonable sample size of the data set (*n *> 100) and for all similarity measures the results show that the fitted model provides a close estimate of the true probability distribution on the set of all patterns (with p-values smaller than 5%). Independent of the number of genetic events the estimate for the induced probability distribution is always statistically significant. Almost all comparisons with the true probability distribution for the different similarity measures have p-values smaller than 0.02.

### Stability of the tree topologies

**Figure 5:**
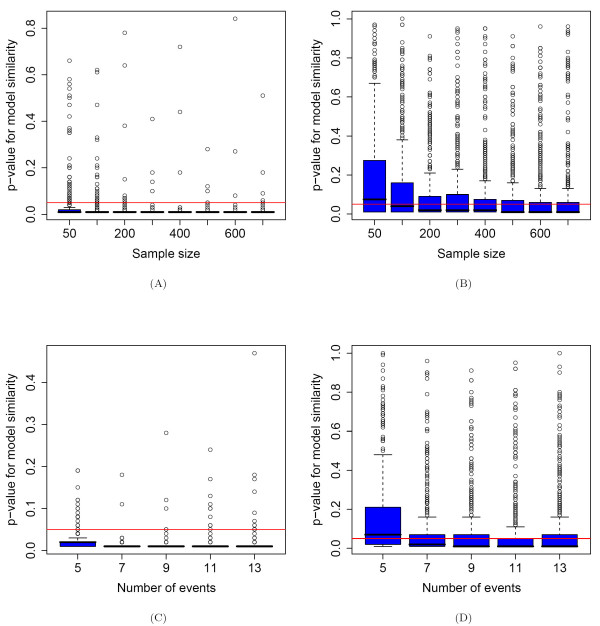
**Stability of tree topology**. A) Significance of the similarity between the tree topologies of the true and the corresponding fitted mixture models for various sample sizes. The mixture models have two components (*K *= 2). The red line depicts the threshold p-value = 0.05. The tree topologies are estimated with high quality. B) Significance analysis as in Figure 5A, but for *K *= 3. For sample sizes larger than 200 the tree topologies are estimated with good quality. C) Significance analysis as in Figure 5A for various numbers of genetic events. The tree topologies are estimated with high quality. D) Significance analysis as in Figure 5C, but for *K *= 3. Except for *l *= 5 genetic events, the estimation of the topologies of the branchings is of good quality.

The topology of the branchings comprising the mixture model is an important model property that determines the order of the genetic events during disease progression. It also establishes the notions of early and late events which are crucial in the waiting time simulation used to calculate the GPS values. We examined the quality of the fitted tree topologies with the simulation setup described in the Methods section. We used the similarity measure given by (6) for comparing the topologies of the tree components between the true and the corresponding fitted model. The boxplots displaying the results for different sample sizes and different numbers of genetic events for *K *= 2 are depicted in Figures 5A and 5C, and the same results for *K *= 3 in Figures 5B and 5D, respectively.

For *K *= 2 tree components (one nontrivial branching) we observe significant similarity between the topology of the trees from the original model and the topology of the trees from the fitted model, independent of the size of the dataset and of the number of genetic events. For *K *= 3 components the results are worse, since two nontrivial branchings in the models make the fitting problem more difficult. As shown by Desper *et al*. [8], using a sufficiently large data sample generated by a mutagenetic tree one can reproduce the original tree with high probability. When there are at least two nontrivial branchings in the mixture model, they have to be estimated with the EM-like algorithm (see [1,10] and the Methods section).

However, as can be seen from Figure 5B, for dataset sizes larger than or equal to 200 the topologies of the mixture components are estimated with good quality for most of the simulation iterations. Also, when varying the number of genetic events (see Figure 5D), the quality of the learned tree topologies does not vary much. This is not true for *l *= 5 genetic events, since this number is not large enough for having two diverse pathways of disease progression in the randomly generated models. For *l *= 5 usually two estimated nontrivial tree components are very similar and many patterns in the dataset can be mapped to both trees with high probability. This makes it very difficult for the clustering algorithm [11] and the EM-like algorithm [10] to correctly separate the data and estimate the true topologies with high significance.

### Stability of the GPS values

**Figure 6:**
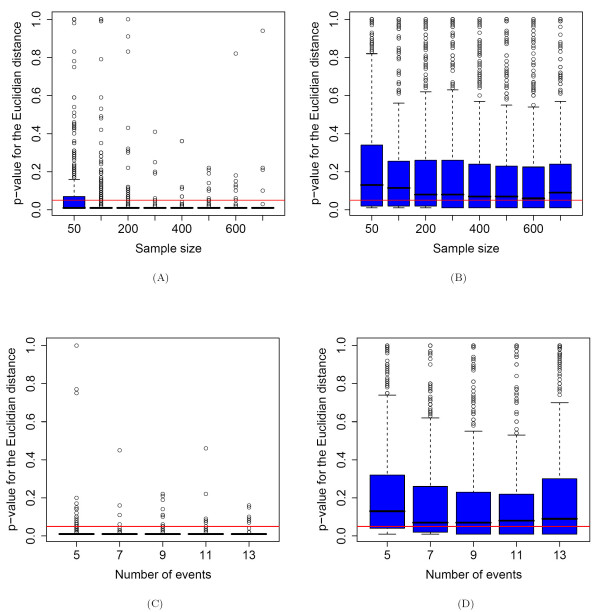
**Stability of GPS**. A) Significance of the similarity between the GPS vectors resulting from the true and its corresponding fitted mixture models for various sample sizes. The mixture models have two components (*K *= 2). The red line depicts the threshold p-value = 0.05. For sample sizes larger than 100 the similarity between the GPS vector estimated from the true model and the one estimated from the fitted model is highly significant. B) Significance analysis as in Figure 6A, but for *K *= 3. The GPS vector estimated from the fitted model is unstable even for large sample sizes used for learning the fitted model. C) Significance analysis as in Figure 6A for various numbers of genetic events. The GPS vectors are estimated with high quality. D) Significance analysis as in Figure 6C, but for *K *= 3. The GPS vector estimated from the fitted model is unstable.

The GPS values [2] are estimated with a waiting time simulation from an underlying mutagenetic trees mixture model based on some additional assumptions (for more details see the Methods section and formula (1)). The simulation study for examining GPS stability was carried out with the standard simulation setup given in the Methods section. The Euclidian distance was used for comparing the estimated GPS vectors associated with the fitted model to the corresponding true GPS vectors. The results for *K *= 2 are given in Figures 6A and 6C, and the results for *K *= 3 are depicted in Figures 6B and 6D.

The simulations with 2-trees mutagenetic mixture models show that for varying sample sizes (larger than 100) and for varying number of genetic events the similarity between the fitted GPS vectors and the corresponding true GPS vectors is highly significant. This is not the case for *K *= 3.

As already mentioned above, for more than one nontrivial tree component in the mixture model, the fitting problem is more complicated since we have incomplete information on which patterns were generated from which branching.

Additionally, the GPS values are highly sensitive to changes in the values of the conditional probabilities assigned to the edges of the branchings, changes in the values of the mixture parameters and modifications in the tree topologies. This can be inferred from the way by which they are calculated (see formula (1) and the Methods section). From the simulation results depicted in Figure 6B it can be seen that the similarity between the GPS vector estimated from the original and the one from the fitted model is not significant at the 5% level for a large portion of the tested models. Increasing the size *n *of the dataset used for estimating the mixture models does not give significant improvements for *n *> 200.

The experimental results from the simulation analysis shown here and in the previous two sections demonstrate that, for *l *= 9 genetic events, datasets with around 200 – 300 samples are sufficiently large for generating a 3-trees mutagenetic mixture model and investigating its features. When varying the number of genetic events for a fixed sample size, it can be seen from Figure 6D that for *l *= 5 genetic events the results are much worse than the rest. As mentioned before, the reason for this behavior is the lack of resulting diversity of the nontrivial model components.

Finally, we compare the boxplots in Figures 6B and 6D from the GPS analysis with the boxplots in the corresponding Figures 5B and 5D from the analysis of the tree topologies. These were calculated from the same simulation runs, i.e. for the same pairs of true and fitted models. It can be observed that when the tree topologies are better reproduced, the similarity between the true and fitted GPS vectors are also higher.

### Bootstrap analysis of GPS values

The stability analysis presented in the preceding paragraphs showed that the GPS value is not a stable feature of the mixture model estimation. We thus performed a *bootstrap *[3] analysis for inspecting the variance of the GPS values resulting from a mutagenetic trees mixture model fitted to the HIV dataset [7].

As depicted in Figure 2, when learning a 2-trees mixture model, the nontrivial component captures the two typical pathways 70 – 219 and 215 – 41 of HIV evolution under zidovudine pressure. We examined the GPS values and their confidence intervals along the edges of the two pathways. In almost all cases, GPS confidence intervals of subsequent patterns in the path are increasing and, if they are overlapping then only to a small extent. Especially the pathway 215 – 41 is stable with typically non-overlapping confidence intervals for subsequent steps in the path.

**Figure 7:**
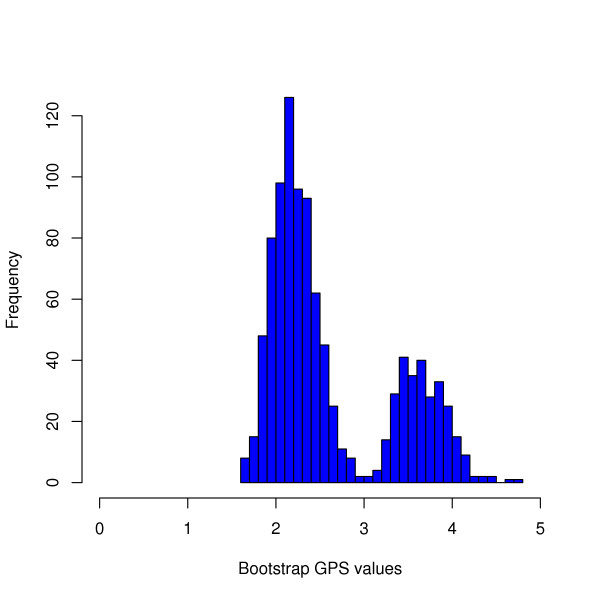
**Histogram of GPS bootstrap distribution**. Histogram of the GPS values for the subset of genetic events {0, 70*R*, 67*N*, 215*F/Y*, 41*L*, 210*W*} estimated from the mutagenetic trees mixture model fitted on 1000 bootstrap samples (bootstrap GPS values). The bootstrap samples are drawn from the HIV dataset.

However, for some patterns the GPS estimation is less reliable. For the pattern *x *= {0, 70*R*, 67*N*, 215*F/Y*, 41*L*, 210*W*}, the 95% confidence interval of the GPS [1.81, 4.04] shows large variability. We observed a bimodal shape of the bootstrap distribution shown in Figure 7. For many bootstrap samples drawn from the HIV data the mixture model is estimated as given in Figure 2. Due to the missing event 219*E/Q *the pattern *x *can only be mapped to the noise component and obtains a GPS value in the interval [1.5, 3.0]. However, for some of the bootstrap samples the event 67*N *is placed after the event 70*R *in the nontrivial tree component of the estimated model. In this case the pattern *x *can also be mapped to the nontrivial tree and its GPS value is larger, i.e. it lies within the interval [3,5]. The low reliability of the GPS value is thus due to the low confidence in the order of occurrence of the events 67*N *and 70*R*.

## Conclusion

We considered the mixture model of mutagenetic trees for modeling disease progression that is characterized by ordered accumulation of permanent genetic changes. First, we improved the model estimation by providing a different starting solution in the EM-like algorithm [1,10]. We introduced new assignments of initial responsibilities of observations with respect to model components. With simulation studies we examined the influence of these initial assignments on the final model estimated on a given dataset. This assessment included the analysis of the quality of different model features like the probability distribution induced by the model, the tree topologies, the GPS, and diversity of the evolutionary paths captured by the model. The study showed that there exists an optimal trade off between desired diversity of the model components and quality of the fitted model.

We evaluated the mutagenetic trees mixture model by performing a detailed analysis of the stability on different levels of the model using different similarity measures. According to this analysis the probability distribution induced by the mixture model can be estimated with high precision. The model also yields a good estimation of the topologies of its tree components.

We observed that the GPS estimated from an underlying fitted mixture model can be sensitive to changes in the tree topologies of the model components and to changes in the conditional probabilities mapped to the edges. With a bootstrap analysis we examined the variability of GPS values and derived corresponding confidence intervals. This helps in determining which GPS values are reliable and can be used for drawing conclusions about the stage of a disease in a specific patient. The sample size was shown to be most critical for the stability of the GPS value. According to the simulation results, for a mutagenetic trees mixture model with *l *= 9 genetic events a sample size of 200 – 300 patients is sufficient for obtaining reasonable estimates.

Since the mutagenetic trees mixture model offers a highly significant estimation of the probability distribution that it induces and also manages to recover the tree topologies with good quality, a future objective is to improve the GPS estimation or to find other ways for using the mixture model in estimating the stage of disease progression. The results of this study are also of interest for other applications of graphical models. It is striking that even when induced probability distributions and topologies of the estimated models are close to those of the true underlying models there is no guarantee that derived scores like the progression along a tree model are highly reliable.

## Methods

In the Methods section we introduce the similarity measures used for comparing mutagenetic trees mixture models and give a detailed description of the simulation setup used in the stability analysis.

### Similarity measures for tree mixture models

The mixture model induces a discrete probability distribution on the set of all possible patterns. In the simulation studies we use three different similarity (distance) measures for exploring the stability of the probability distributions induced by the fitted mixture models, namely the *L*_1 _distance, the Cosine distance, and the Kullback-Leibler divergence.

In the following, we present similarity measures for tree topologies that are needed both for comparing two different mixture models and for comparing the nontrivial components within a single mixture model. Yin *et al*. [12] proposed to use the difference of the maximum number of outgoing edges as a dissimilarity measure of the topologies of two directed branchings. In the following we present two other more sophisticated similarity measures for tree topologies.

Let *M*_1 _and *M*_2 _be *K*-mutagenetic trees mixture models (*K *≥ 2) on a set of *l *genetic events. In order to compare the topologies of the trees of the two mixture models, we first form pairs of similar components. In other words, to each branching in *M*_1 _we associate the most similar branching from *M*_2_, such that every branching from *M*_2 _corresponds to exactly one branching in *M*_1_. This is the cost minimizing assignment problem which can be solved by the *Hungarian algorithm *[13] in polynomial time (*O*((*K *– 1)^3^)). The noise components in the mixture models have identical topology, so we leave them out.

Let $M_{i}^{\left(j\right)}$, *i *∈ {1, 2}, *j *∈ {1,...,*K*} denote the *i*-th branching of the mixture model *M*_*j *_and let :Π {2,...,*K*} → {2,...,*K*}; denote all possible permutations of the set {2,...,*K*}. We start by building a cost matrix:

$$S={\left(s_{ij}\right)}_{\begin{array}{l} 1\leq i\leq K-1,\\ 1\leq j\leq K-1 \end{array}}$$

where *s*_*ij *_is the edge difference between tree component (*i *+ 1) of *M*_1 _and tree component (*j *+ 1) of *M*_2 _formally denoted by e.dif ($M_{i+1}^{\left(1\right)},M_{j+1}^{\left(2\right)}$)

Afterwards, we apply the Hungarian algorithm [13] on the cost matrix **S **to find the assignment Π_match _that minimizes the dissimilarity of the trees of the two mixture models. This is given by the formula

(4) $$\Pi_{\text{match}}=\underset{\Pi }{\arg\min}\sum_{k=1}^{K}\text{e}\text{.dif}(M_{k}^{\left(1\right)},M_{\Pi (k)}^{\left(2\right)}),$$

where the minimum is taken over all possible permutations Π of the set {2,...,*K*}. Hence, Π_match _defines (*K *– 1) similarity pairs of tree components of the two models *M*_1 _and *M*_2 _by using the number of different edges as a dissimilarity measure. In what follows, we use these pairs for the introduction of two similarity measures for comparing the mixture models *M*_1 _and *M*_2_.

One straightforward way to measure the difference (or similarity) of the tree topologies of the mixture models is to simply add the number of different edges of the similarity pairs. Considering (4), this is formally given by:

(5) $$\text{Dif}(M_{1},M_{2})=\sum_{k=2}^{K}\text{e}\text{.dif}(M_{k}^{\left(1\right)},M_{\Pi_{\text{match}}(k)}^{\left(2\right)}).$$

We divide the sum in (5) by the maximum possible number of different edges (*K *– 1)·(*l *– 1), in order to obtain a normalized number inside the interval [0, 1].

The topology of the components of the mixture models should capture the order in which the genetic changes accumulate during disease progression. It is a significant difference, if an event appears very early in one tree and very late in another tree. We thus define a similarity measure that also takes into account the levels (depths) of the events in the tree. We associate a *level vector *to each nontrivial tree component of the mixture models. In our setting, the *level vectors *have length*l *(the number of genetic events), where each component corresponds to a specific genetic event and gives the level (depth) of that event in the considered tree topology. For comparison purposes the order of the vector components has to be fixed in all level vectors.

The enhanced topology distance measure is constructed by adding to each edge difference of a similarity pair in formula (5) the *L*_1 _distance of the level vectors of the trees creating the similarity pair. Let level.vec(*T |T *∈ *M*) denote the level vector associated with the events of the tree component *T *from the mixture model *M*. The similarity measure is then given by:

(6) $$\text{Dif}(M_{1},M_{2})=\sum_{k=2}^{K}\left(\text{e}\text{.dif}\left(M_{i}^{\left(1\right)},M_{\Pi_{\text{match}}(i)}^{\left(2\right)}\right)+L_{1}\left(\text{level}\text{.vec}\left(M_{i}^{\left(1\right)}\right),\text{level}\text{.vec}\left(M_{\Pi_{\text{match}}(i)}^{\left(2\right)}\right)\right)\right).$$

Since the level vectors reflect the order of the genetic events in the branchings, this similarity measure is more model-specific when it comes to comparing topologies of two mutagenetic trees mixture models. This is important for assessing the quality of the mixture models and thus (6) is used in the stability analysis.

The similarity measure introduced in (6) was constructed for comparing the topologies of the components of two mixture models. It can also be used for comparing the topologies of the nontrivial branchings in a single mutagenetic trees mixture model. This enables analyzing the diversity of the paths of ordered genetic events in disease progression captured in each branching of a given mixture model. In this case, the procedure of forming similarity pairs is not needed. The diversity of the nontrivial tree components of a mixture model can be quantitatively expressed by the sum of the comparisons of all nontrivial tree components against each other (all different sets of two distinct trees).

### Simulation setup for stability analysis

The mutagenetic trees mixture model can be characterized by many different features like the probability distributions induced by the model, the number of tree components, the topologies of the tree components, the GPS values, and so on. This makes the model rather complex, so grasping the essence of its quality and stability demands simulated data from artificial mixture models created uniformly at random from topology space (random models). Having defined true models we can inspect the quality of the fitting procedure by comparing the different features characterizing the true and the fitted mixture models and by estimating their stability. The following simulation setup is similar to the experimental design in [14] and the simulation setup in [12].

The simulation procedure consists of a sufficient number of iterations, where different similarity measures are computed and later used for examining the stability on the different levels of the mixture model. The procedure is given as follows:

1. For a fixed number of tree components *K *and a given number of genetic events *l *draw a *true *mixture model *M *at random.

• The first component *T*_1 _is a noise component with star topology.

• The nontrivial tree components *T*_2_,...,*T*_*K *_are random trees sampled uniformly from topology space. Cayley's theorem [15] states that there are *l*^(*l*-2) ^distinct labeled trees on *l *vertices. Moreover, Prüfer in [16] proved that a labeled tree on *l *vertices can be uniquely encoded as an (*l*-2) tuple of vertex labels. Hence, a random tree can be sampled by generating a random (*l*-2) tuple of vertex labels (the set {1,...,*l*}) and considering it as the Prüfer code of a labeled tree (see [12]).

• The edge weights of the trees are drawn uniformly at random from the interval [0.2, 0.8].

• The tree weights (mixture parameters) are set as follows: *α*_1 _= 0.05 (5% noise) and *α*_*k *_= 0.95/(*K *– 1), where 2 ≤ *k *≤ *K*.

2. Draw a sample *D *(a certain number *n *of patterns) from the true mixture model *M*.

3. Fit a *K*-trees mutagenetic mixture model *M*_*fit *_to the sample *D*.

4. Compare different model features (GPS, distributions, tree topologies) of the fitted model *M*_*fit *_with the corresponding features of the true model *M *by computing the values of the appropriate similarity measures *(true similarities)*.

5. Generate 100 random models and compare their model features with the corresponding features of the true model *M *by computing the appropriate similarity measures *(random similarities)*.

6. Use the values of the *random similarities *to calculate the *p-values *for the *true similarities*. The *p-value *is the proportion of random similarities that are equal to or smaller than the corresponding true similarities.

For the analysis of the influence of the initial clustering in the EM-like algorithm, we compare two tree mixture models, namely the initial one constructed directly from the initial assignments and the final one obtained when running the entire EM-like algorithm. The procedure for one simulation iteration is given as follows:

1. Draw a *true *mixture model *M *uniformly at random with *K *= 3 branchings on *l *= 9 genetic events.

2. Draw a sample *D *from the model *M *with 500 patterns.

3. Fit a K-trees mixture model *M*_*init *_to the dataset *D *based only on the clustering results. In other words, we build the *K *tree components *T*_1_,...,*T*_*K *_with the tree reconstruction algorithm [8,9] from all patterns weighted with their initial responsibilities (resulting from the clustering) and we compute the mixture parameters *α*_1_,...,*α*_K_.

4. Fit a *K*-trees mixture model *M*_*fit *_by using the EM-like learning algorithm [1].

5. Compare the topologies of the nontrivial tree components in the mixture models *M*_*init *_and *M*_*fit *_by using the similarity measure (6) for comparing tree topologies.

## Authors' contributions

JB implemented the algorithms, computed and interpreted the results and drafted the manuscript. TL contributed to the design of the study and to the interpretation of results. JR conceived and designed the study and contributed to the computation and interpretation of results. All authors contributed to the writing of the manuscript and read and approved the final version.
